# Severe Euvolemic Hyponatremia Consistent With Syndrome of Inappropriate Antidiuretic Hormone Secretion (SIADH) During Localized Herpes Zoster in an Older Adult

**DOI:** 10.7759/cureus.111926

**Published:** 2026-07-01

**Authors:** Abinaya Rajendran, Dhayanithi Dhayalan, Sreedhar Adapa

**Affiliations:** 1 Internal Medicine, Pondicherry Institute of Medical Sciences, Kalapet, IND; 2 Nephrology, Adventist Health Community Care, Hanford, USA; 3 Nephrology, Kaweah Health Medical Center, Visalia, USA

**Keywords:** acyclovir therapy, herpes zoster reactivation, herpes zoster virus, severe siadh, site of infection, syndrome of inappropriate secretion of antidiuretic hormone (siadh)

## Abstract

Syndrome of inappropriate antidiuretic hormone secretion (SIADH) is an uncommon but recognized complication of herpes zoster (varicella-zoster virus, VZV) infection, and diagnostic uncertainty may arise when hyponatremia develops after antiviral therapy is initiated. We report an 89-year-old patient with hypertension, chronic kidney disease stage 3, hyperlipidemia, and prior left nephrectomy for renal cell carcinoma who presented with right neck and upper arm pain, weakness, and a unilateral dermatomal vesicular eruption consistent with herpes zoster. Oral acyclovir was started at 800 mg five times daily. During hospitalization, serum sodium declined from 136 mEq/L to a nadir of 123 mEq/L despite discontinuation of hydrochlorothiazide. The patient remained clinically euvolemic, with urine sodium of 134 mEq/L and urine osmolality of 570 mOsm/kg, supporting SIADH. Thyroid-stimulating hormone and morning cortisol were normal. Fluid restriction < 1.2 L/day was ineffective. As the patient became symptomatic, and hypertonic saline (with removal of fluid restriction) produced no improvement, a single dose of tolvaptan produced rapid correction of serum sodium and improvement in mentation. The patient was subsequently maintained on sodium chloride tablet supplementation, fluid restriction, and a high-protein diet, with serum sodium improving to 135-136 mEq/L. This case describes severe hypotonic euvolemic hyponatremia meeting biochemical criteria for SIADH in the setting of localized herpes zoster, after consideration of concurrent hydrochlorothiazide exposure and temporally associated oral acyclovir.

## Introduction

Herpes zoster (varicella-zoster virus, VZV) is caused by reactivation of the latent VZV and classically presents with a painful unilateral dermatomal eruption. Although dermatologic and neuropathic manifestations are well recognized, the syndrome of inappropriate anti-diuretic hormone (SIADH) is an uncommon complication and may be overlooked when more common causes of hyponatremia are present. Diagnostic uncertainty becomes greater when antiviral therapy is started near the time of sodium decline, because medication-associated hyponatremia may also be considered. We report a case of biochemical SIADH occurring during localized cervical dermatomal herpes zoster in an older adult, with emphasis on the diagnostic challenge posed by concurrent thiazide exposure and oral acyclovir therapy [1-6].

## Case presentation

**Figure 1:**
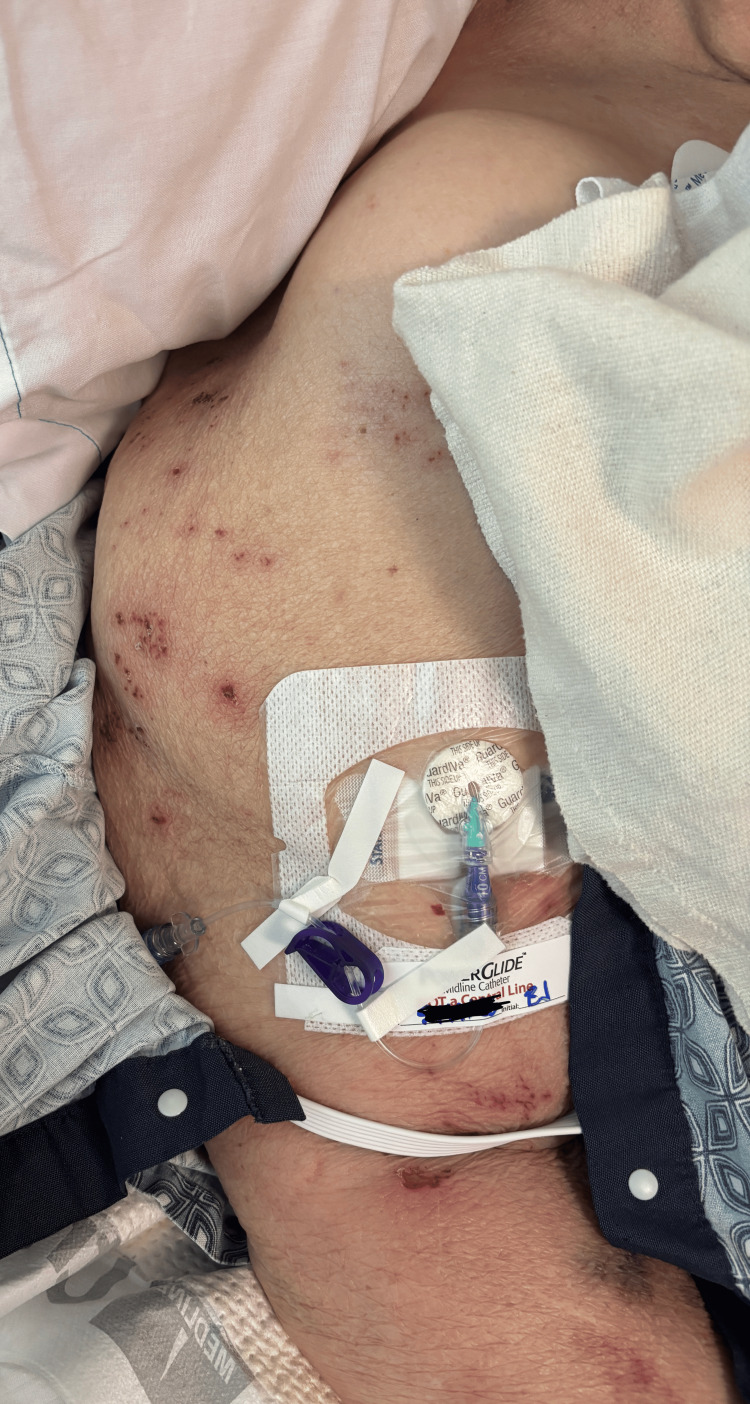
Maculopapular and vesicular eruption of shingles involving C4-C6 dermatomes in different stages

An 89-year-old patient with hypertension, chronic kidney disease stage 3, hyperlipidemia, and prior left nephrectomy for renal cell carcinoma in 1989 presented to the emergency department on Day 5 with right neck pain, right upper arm pain, and weakness after symptom onset on Day 1. Vital signs were within normal limits on presentation. Cardiovascular, respiratory, and abdominal examinations were unremarkable. There was no jugular venous distension, mucous membranes were moist, and there was no peripheral edema. Skin examination demonstrated a unilateral dermatomal maculopapular and vesicular eruption with lesions in different stages, including pustular and crusted lesions, consistent with herpes zoster (Figure 1). Neurologic examination showed 2/5 motor strength in the right upper extremity in a C4-C5 and C5-C6 distribution. There were no sensory disturbances on the examination. 

Home medications included losartan 25 mg daily, pantoprazole 20 mg daily, atorvastatin 80 mg daily, and hydrochlorothiazide 25 mg daily. She takes no over-the-counter (OTC) medications. Computed tomography of the head showed no acute intracranial abnormality. Magnetic resonance imaging of the cervical spine demonstrated uncovertebral spurring causing severe bilateral neural foraminal impingement at C4-C5 and moderate left with severe right neural foraminal impingement at C5-C6, which offered a structural explanation for the right arm weakness. Oral acyclovir was started on Day 5 at 800 mg five times daily for herpes zoster. The patient's pain was 1/10 in severity, for which she required acetaminophen 650 mg once during presentation, after which she did not report any further pain.

**Figure 2:**
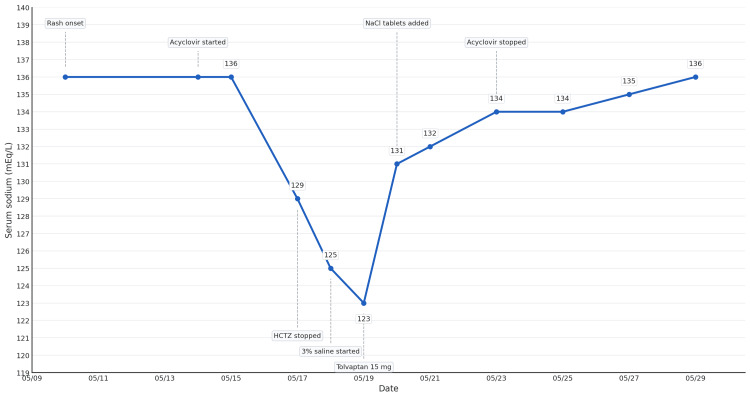
Serum sodium levels with clinical events HCTZ: hydrochlorothiazide, NaCl: sodium chloride

Serum sodium was 136 mEq/L on Day 6, and it kept dropping, ultimately reaching a nadir of 123 mEq/L. The patient had hypotonic hyponatremia and remained clinically euvolemic. Urine sodium was 134 mEq/L, and urine osmolality was 570 mOsm/kg, findings consistent with SIADH in the appropriate clinical setting (Figure 2). A basic metabolic panel obtained on Day 9 showed sodium of 125 mEq/L, potassium of 3.8 mEq/L, chloride of 89 mEq/L, bicarbonate of 25 mEq/L, glucose of 90 mg/dL, blood urea nitrogen of 23 mg/dL, creatinine of 0.9 mg/dL, estimated glomerular filtration rate of 59 mL/min/1.73 m2, serum osmolarity of 270 mOsm/kg, and calcium of 9.3 mg/dL (Table 1). 

**Table 1:** Lab values from Day 9 eGFR: estimated glomerular filtration rate, TSH: thyroid-stimulating hormone

Lab	Value	Reference range
Sodium	125 mEq/L	136-142 mEq/L
Potassium	3.8 mEq/L	3.5-5.0 mEq/L
Chloride	89 mEq/L	96-106 mEq/L
Bicarbonate	25 mEq/L	22-26 mEq/L
Glucose	90 mg/dL	70-110 mg/dL
Blood urea nitrogen	23 mg/dL	8-23 mg/dL
Creatinine	0.9 mg/dL	0.6-1.2 mg/dL
eGFR	59 mL/min/1.73 m^2^	>90 mL/min/1.73 m^2^
Calcium	9.3 mg/dL	8.2-10.2 mg/dL
TSH	2.8 mIU/L	0.4-4.0 mIU/L
Morning cortisol	19.9 mcg/dL	5-25 mcg/dL
Urine sodium	134 mEq/L	
Urine osmolality	570 mOsm/kg	
Serum Osmolarity	270 mOsm/kg	275-295 mOsm/kg
Uric acid	3.8 mg/dL	3-8 mg/dL

Hydrochlorothiazide was discontinued on Day 8, and the patient was started on < 1.2 L fluid restriction per day, but serum sodium continued to decline. As hyponatremia worsened, the patient developed mild encephalopathy and generalized weakness. Fluid restriction was removed, and 3% saline at 30 mL/hour was started, which did not produce any improvement either. Given persistent symptomatic hyponatremia despite initial therapy, tolvaptan of 15 mg once was administered with close serial sodium monitoring, after which serum sodium increased to 131 mEq/L within 24 hours and mental status improved. Sodium correction remained within the institutionally accepted safety threshold, and no clinical features of osmotic demyelination developed. The patient was subsequently managed with sodium chloride tablets 1 g twice daily, a high-protein diet, and fluid restriction. Serum sodium improved and stabilized at 136 mEq/L by Day 20 (Figure 2).

Evaluation for alternative endocrine causes of hyponatremia was unrevealing. Thyroid-stimulating hormone was 2.8, and morning cortisol was 19.9 (Table 1). The patient completed a 10-day course of oral acyclovir on Day 14.

## Discussion

This case is most consistent with SIADH occurring in the setting of VZV in an older adult. The patient had hypotonic hyponatremia with a typical dermatomal eruption, remained clinically euvolemic, and demonstrated inappropriately elevated urine sodium and urine osmolality, all of which support SIADH.

Herpes zoster-associated SIADH remains an uncommon but increasingly recognized complication of VZV reactivation. The pathophysiology of SIADH associated with localized herpes zoster remains unclear. One proposed explanation is that the VZV may disrupt the neural pathways that regulate antidiuretic hormone (ADH) secretion. Herpes zoster remains latent in the sensory neurons of the dorsal root ganglia, which transmit signals related to pain, temperature, pressure, vibration, and proprioception. After reactivation, the virus spreads along the affected sensory axons to the corresponding dermatome and may also extend centripetally toward the dorsal columns of the spinal cord. Because some peripheral osmoreceptive signals also travel through the dorsal root ganglia before reaching the spinal cord and central nervous system, herpes zoster infection of these neurons could interfere with osmoregulation and ADH control. Earlier reports described only a few cases, and subsequent publications have continued to add isolated case reports, suggesting that this entity may be underrecognized in routine practice. Most reported cases involve the V1 dermatome, followed by thoracic dermatomes. To date, only two cases involving the cervical dermatome have been reported, based on a 2018 case review [1-4].

Hydrochlorothiazide and oral acyclovir were plausible contributors. Although there are a few case reports of acyclovir-associated SIADH, most have involved intravenous administration and improvement after drug discontinuation. In our patient, the temporal association with oral acyclovir raised the possibility of a drug effect, but the persistence of hyponatremia after drug discontinuation was less supportive of acyclovir as the sole cause, although causality cannot be excluded [5,6]. Similarly, recent thiazide exposure may have contributed to or amplified the hyponatremic process. Pain is an important consideration. However, the patient reported only minimal pain on presentation, which improved after a single dose of acetaminophen, and she denied any subsequent pain. Therefore, pain is less likely to be the underlying cause.

This case highlights the importance of considering herpes zoster in the differential diagnosis of new euvolemic hyponatremia, especially when a classic dermatomal rash is present. It also underscores the need to interpret temporally associated medications and concurrent abnormalities carefully so that the principal driver of hyponatremia is not overlooked.

## Conclusions

This case describes severe euvolemic hypotonic hyponatremia consistent with SIADH occurring during localized herpes zoster in an older adult. Herpes zoster may have been an important precipitating factor; however, recent hydrochlorothiazide exposure, oral acyclovir, pain, and acute illness may have contributed. Clinicians should consider herpes zoster among potential triggers of SIADH while systematically evaluating alternative and coexisting causes.
